# *rac*-(*S*,*S*)-Bis(1-ferrocenylbut-3-en­yl) ether

**DOI:** 10.1107/S1600536812048064

**Published:** 2012-12-08

**Authors:** Hao-Jun Xie, Chun-Zheng Zhao, Jun Sun, Si Chen, Jian-Jun Wang

**Affiliations:** a College of Chemistry, Chemical Engineering and Materials Science, Soochow University, Suzhou, Jiangsu 215123, People’s Republic of China

## Abstract

The title complex, [Fe_2_(C_5_H_5_)_2_(C_18_H_20_O)], formed by dehydration of 1-ferrocenylbut-3-en-1-ol, crystallizes as a racemic compound. The central C—O—C fragment, in which the C atoms are the chiral centers, is characterized by an angle of 116.26 (10)° at the O atom. One ferrocene group shows a staggered conformation whereas the other shows an eclipsed conformation.

## Related literature

For general information on ferrocenyl ethers, see: Ferguson *et al.* (1996 ▶); Matković-Čalogović *et al.* (1993 ▶); Gasser *et al.* (2007 ▶). For applications of dinuclear ferrocenyl derivatives, see: Gao *et al.* (2011 ▶); Locke *et al.* (2001 ▶).
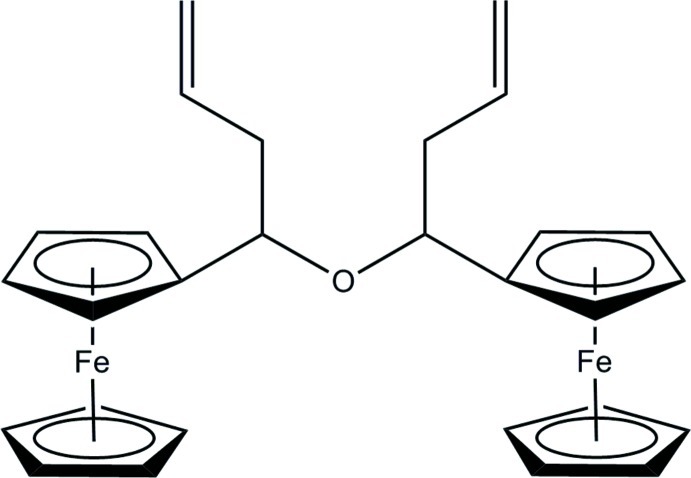


## Experimental

### 

#### Crystal data

[Fe_2_(C_5_H_5_)_2_(C_18_H_20_O)]
*M* *_r_* = 494.22
Triclinic, 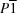

*a* = 9.7865 (15) Å
*b* = 9.8274 (15) Å
*c* = 12.1816 (19) Å
α = 99.405 (2)°
β = 94.976 (2)°
γ = 101.657 (2)°
*V* = 1123.3 (3) Å^3^
*Z* = 2
Mo *K*α radiation
μ = 1.31 mm^−1^
*T* = 150 K
0.50 × 0.25 × 0.25 mm

#### Data collection

Bruker APEXII CCD diffractometer
Absorption correction: multi-scan (*SADABS*; Bruker, 2007 ▶) *T*_min_ = 0.561, *T*_max_ = 0.736
7657 measured reflections
5352 independent reflections
4759 reflections with *I* > 2σ(*I*)
*R*_int_ = 0.014

#### Refinement

*R*[*F* ^2^ > 2σ(*F* ^2^)] = 0.027
*wR*(*F* ^2^) = 0.100
*S* = 0.81
5352 reflections
280 parameters
H-atom parameters constrained
Δρ_max_ = 0.36 e Å^−3^
Δρ_min_ = −0.37 e Å^−3^

### 

Data collection: *APEX2* (Bruker, 2007 ▶); cell refinement: *SAINT* (Bruker, 2007 ▶); data reduction: *SAINT*; program(s) used to solve structure: *SHELXS97* (Sheldrick, 2008 ▶); program(s) used to refine structure: *SHELXL97* (Sheldrick, 2008 ▶); molecular graphics: *SHELXTL* (Sheldrick, 2008 ▶); software used to prepare material for publication: *SHELXTL*.

## Supplementary Material

Crystal structure: contains datablock(s) I, global. DOI: 10.1107/S1600536812048064/bh2465sup1.cif

Structure factors: contains datablock(s) I. DOI: 10.1107/S1600536812048064/bh2465Isup2.hkl

Additional supplementary materials:  crystallographic information; 3D view; checkCIF report

## Figures and Tables

**Fig. 1.**
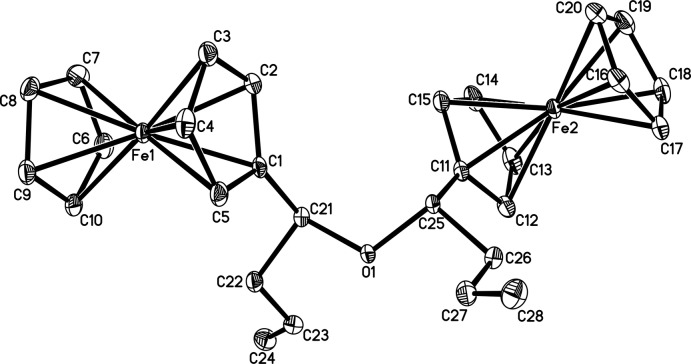
The molecular structure of the title complex, showing 30% probability displacement ellipsoids. All H atoms have been omitted for clarity.

**Fig. 2.**
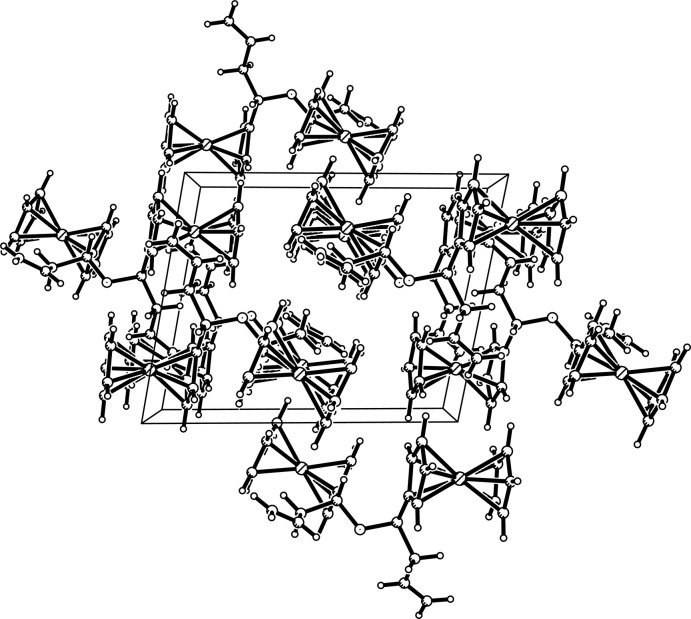
The crystal structure of the title compound.

## supplementary crystallographic information

### Comment

Due to their high iron content, bisferrocenyl derivatives have a wider range of applications in high burning rate catalysts than mononuclear ferrocenyl ones (Gao *et al.*, 2011; Locke *et al.*, 2001). Most of bisferrocenyl ethers are synthesized from alcohols with H_2_SO_4_ or Al_2_O_3_ as catalysts in the environment of high temperature and vacuum (Ferguson *et al.*, 1996; Matković-Čalogović *et al.*, 1993). As part of our ongoing program, the title compound was prepared from 1-ferrocenyl-3-buten-1-ol and dicyclohexylcarbodiimide (DCC), with 4-dimethylaminopyridine (DMAP) as catalyst, at room temperature, and studied by X-ray crystallography. The complex crystallizes with a triclinic unit cell in the *P*1 space group. A view of the asymmetric unit is given in Fig. 1 and the crystal structure in Fig. 2. Structure solution and refinement showed that the racemic form was crystallized with both chiral centers having the same configuration.

The principal molecular features are the structure of the central C—O—C bridge and the conformations of the ferrocene fragments. The C—O bond lengths are 1.4399 (15) and 1.4430 (15) Å, and the C—O—C angle is 116.26 (10)°, while this angle is found between 113.7 (9) and 112.8 (9)° in the crystal structure of bis(ferrocenylmethyl)ether (Gasser *et al.*, 2007). The cyclopentadienyl rings are twisted from the eclipsed conformation: the mean torsion angles C*n*—*Cg*1—*Cg*2—*Cm* and C*p*—*Cg*3—*Cg*4—C*q* (*n* = 1···5, *m* = *n* + 5; *p* = 11···15, *q* = *p* + 5; the *Cg* pseudoatoms are the centroids of the four rings), for the ferrocene groups defined by Fe1 and Fe2, are 29.3 (1) and 6.1 (1)°, respectively.

### Experimental

A solution of 1-ferrocenyl-3-buten-1-ol (5.862 g, 34.89 mmol), DCC (9.597 g, 46.52 mmol) and DMAP (2.842 g, 23.26 mmol) in CH_2_Cl_2_ (200 ml) was stirred at room temperature for 36 h. The solution was filtered off and the filtrate was washed with CH_2_Cl_2_. The organic phases were combined and dried to give a viscous yellow oil, which was chromatographed over a column of silica gel using petroleum ether as the eluent. Yellow crystals of the title compound were obtained by slow evaporation of a solution in dichloromethane/petroleum ether (60–90°C). ^1^H NMR (400 MHz, CDCl_3_) δ 6.17–5.79 (m, 2H, H23A and H27A), 5.26–4.96 (m, 4H, H24A–H24B and H28A–H28B), 4.37 (s, 2H, H21A and H25A), 4.46–3.90 (m, 18H, H2–H10 and H12–H20), 2.84–2.46 (m, 4H, H22A–H22B and H26A–H26B). HRMS (ESI): calcd for C_28_H_30_Fe_2_O: 494.0990, found 494.0981.

### Refinement

H atoms were placed in calculated positions and thereafter treated as riding atoms, with C—H = 0.98 (Cp rings and methine CH), 0.93 (vinyl CH), and 0.97 Å (methylene CH_2_). Isotropic displacement parameters for H atoms were calculated as *U*_iso_(H) = 1.2*U*_eq_(carrier C).

### Crystal data

**Table:**

[Fe_2_(C_5_H_5_)_2_(C_18_H_20_O)]	*Z* = 2
*M**_r_* = 494.22	*F*(000) = 516
Triclinic, *P*1	*D*_x_ = 1.461 Mg m^−^^3^
Hall symbol: -P 1	Mo *K*α radiation, λ = 0.71073 Å
*a* = 9.7865 (15) Å	Cell parameters from 4332 reflections
*b* = 9.8274 (15) Å	θ = 3.0–28.3°
*c* = 12.1816 (19) Å	µ = 1.31 mm^−^^1^
α = 99.405 (2)°	*T* = 150 K
β = 94.976 (2)°	Block, yellow
γ = 101.657 (2)°	0.50 × 0.25 × 0.25 mm
*V* = 1123.3 (3) Å^3^	

### Data collection

**Table:**

Bruker APEXII CCD diffractometer	5352 independent reflections
Radiation source: fine-focus sealed tube	4759 reflections with *I* > 2σ(*I*)
Graphite monochromator	*R*_int_ = 0.014
φ and ω scans	θ_max_ = 28.2°, θ_min_ = 2.2°
Absorption correction: multi-scan (*SADABS*; Bruker, 2007)	*h* = −9→12
*T*_min_ = 0.561, *T*_max_ = 0.736	*k* = −13→8
7657 measured reflections	*l* = −16→15

### Refinement

**Table:**

Refinement on *F*^2^	Primary atom site location: structure-invariant direct methods
Least-squares matrix: full	Secondary atom site location: difference Fourier map
*R*[*F*^2^ > 2σ(*F*^2^)] = 0.027	Hydrogen site location: inferred from neighbouring sites
*wR*(*F*^2^) = 0.100	H-atom parameters constrained
*S* = 0.81	*w* = 1/[σ^2^(*F*_o_^2^) + (0.1*P*)^2^] where *P* = (*F*_o_^2^ + 2*F*_c_^2^)/3
5352 reflections	(Δ/σ)_max_ = 0.001
280 parameters	Δρ_max_ = 0.36 e Å^−^^3^
0 restraints	Δρ_min_ = −0.37 e Å^−^^3^
0 constraints	

### Fractional atomic coordinates and isotropic or equivalent isotropic displacement parameters (Å^2^)

**Table:**

	*x*	*y*	*z*	*U*_iso_*/*U*_eq_	
Fe1	0.20476 (2)	0.792940 (19)	0.468598 (15)	0.01300 (8)	
Fe2	−0.30704 (2)	0.791879 (19)	−0.022770 (16)	0.01411 (8)	
O1	−0.18694 (10)	0.57498 (10)	0.25064 (7)	0.0148 (2)	
C1	0.04463 (15)	0.70792 (14)	0.33824 (10)	0.0142 (3)	
C2	0.13856 (16)	0.82818 (16)	0.31344 (12)	0.0169 (3)	
H2A	0.1124	0.9149	0.2978	0.020*	
C3	0.27661 (16)	0.80081 (17)	0.31594 (12)	0.0196 (3)	
H3A	0.3612	0.8648	0.3019	0.023*	
C4	0.26917 (17)	0.66318 (18)	0.34216 (13)	0.0202 (3)	
H4A	0.3479	0.6162	0.3497	0.024*	
C5	0.12676 (15)	0.60656 (15)	0.35574 (11)	0.0172 (3)	
H5A	0.0913	0.5141	0.3752	0.021*	
C6	0.13881 (17)	0.91467 (17)	0.59802 (13)	0.0207 (3)	
H6A	0.0536	0.9525	0.5942	0.025*	
C7	0.27384 (17)	0.98277 (15)	0.57716 (12)	0.0217 (3)	
H7A	0.2978	1.0758	0.5558	0.026*	
C8	0.36802 (16)	0.89279 (16)	0.59166 (12)	0.0204 (3)	
H8A	0.4681	0.9128	0.5822	0.024*	
C9	0.29131 (17)	0.76805 (17)	0.62240 (12)	0.0201 (3)	
H9A	0.3292	0.6871	0.6378	0.024*	
C10	0.14978 (15)	0.78253 (15)	0.62714 (11)	0.0184 (3)	
H10A	0.0730	0.7128	0.6460	0.022*	
C11	−0.25795 (15)	0.71814 (15)	0.12099 (11)	0.0158 (3)	
C12	−0.40690 (15)	0.70386 (16)	0.09872 (11)	0.0184 (3)	
H12A	−0.4777	0.6149	0.0860	0.022*	
C13	−0.43463 (17)	0.84011 (18)	0.09606 (12)	0.0236 (3)	
H13A	−0.5275	0.8613	0.0819	0.028*	
C14	−0.30363 (19)	0.94001 (17)	0.11764 (13)	0.0241 (3)	
H14A	−0.2905	1.0422	0.1209	0.029*	
C15	−0.19508 (16)	0.86556 (15)	0.13278 (11)	0.0194 (3)	
H15A	−0.0941	0.9079	0.1481	0.023*	
C16	−0.22515 (19)	0.68955 (18)	−0.15448 (13)	0.0215 (3)	
H16A	−0.1663	0.6204	−0.1501	0.026*	
C17	−0.37461 (17)	0.65922 (17)	−0.17461 (12)	0.0214 (3)	
H17A	−0.4367	0.5651	−0.1864	0.026*	
C18	−0.41885 (18)	0.78905 (18)	−0.17327 (12)	0.0231 (3)	
H18A	−0.5162	0.7998	−0.1849	0.028*	
C19	−0.29629 (18)	0.89995 (16)	−0.15316 (12)	0.0231 (3)	
H19A	−0.2945	1.0009	−0.1478	0.028*	
C20	−0.17646 (17)	0.83925 (17)	−0.14101 (12)	0.0228 (3)	
H20A	−0.0781	0.8911	−0.1258	0.027*	
C21	−0.11241 (14)	0.68741 (14)	0.33961 (11)	0.0143 (3)	
H21A	−0.1412	0.7754	0.3311	0.017*	
C22	−0.16178 (15)	0.64072 (15)	0.44594 (11)	0.0168 (3)	
H22A	−0.1355	0.5520	0.4522	0.020*	
H22B	−0.1133	0.7106	0.5104	0.020*	
C23	−0.31686 (15)	0.62190 (15)	0.44919 (12)	0.0185 (3)	
H23A	−0.3767	0.5692	0.3865	0.022*	
C24	−0.37406 (17)	0.67602 (17)	0.53626 (13)	0.0242 (3)	
H24A	−0.3169	0.7292	0.6001	0.029*	
H24B	−0.4713	0.6608	0.5334	0.029*	
C25	−0.18233 (14)	0.60277 (14)	0.13816 (10)	0.0139 (3)	
H25A	−0.0842	0.6312	0.1248	0.017*	
C26	−0.25030 (14)	0.46036 (14)	0.06301 (11)	0.0165 (3)	
H26A	−0.3450	0.4296	0.0808	0.020*	
H26B	−0.2571	0.4725	−0.0146	0.020*	
C27	−0.17049 (16)	0.34783 (16)	0.07565 (12)	0.0203 (3)	
H27A	−0.1437	0.3350	0.1477	0.024*	
C28	−0.13596 (16)	0.26558 (16)	−0.00995 (13)	0.0235 (3)	
H28A	−0.1614	0.2761	−0.0829	0.028*	
H28B	−0.0862	0.1973	0.0029	0.028*	

### Atomic displacement parameters (Å^2^)

**Table:**

	*U*^11^	*U*^22^	*U*^33^	*U*^12^	*U*^13^	*U*^23^
Fe1	0.01460 (12)	0.01352 (13)	0.00977 (12)	0.00283 (9)	−0.00164 (8)	0.00097 (8)
Fe2	0.01641 (13)	0.01587 (13)	0.00999 (12)	0.00418 (9)	−0.00097 (8)	0.00283 (8)
O1	0.0184 (5)	0.0151 (5)	0.0085 (4)	−0.0002 (4)	−0.0016 (3)	0.0023 (3)
C1	0.0170 (6)	0.0169 (6)	0.0073 (6)	0.0030 (5)	−0.0012 (5)	0.0007 (5)
C2	0.0188 (7)	0.0196 (7)	0.0113 (6)	0.0021 (5)	−0.0004 (5)	0.0038 (5)
C3	0.0178 (7)	0.0272 (8)	0.0125 (6)	0.0031 (6)	0.0005 (5)	0.0033 (5)
C4	0.0193 (7)	0.0255 (8)	0.0147 (7)	0.0081 (6)	−0.0017 (5)	−0.0019 (6)
C5	0.0216 (7)	0.0160 (6)	0.0117 (6)	0.0036 (5)	−0.0030 (5)	−0.0010 (5)
C6	0.0239 (8)	0.0221 (7)	0.0144 (7)	0.0071 (6)	−0.0004 (6)	−0.0021 (5)
C7	0.0311 (8)	0.0147 (6)	0.0159 (7)	0.0009 (6)	0.0007 (6)	−0.0011 (5)
C8	0.0187 (7)	0.0225 (7)	0.0151 (7)	−0.0005 (6)	−0.0028 (5)	−0.0013 (5)
C9	0.0223 (7)	0.0216 (7)	0.0143 (7)	0.0037 (6)	−0.0047 (5)	0.0027 (5)
C10	0.0200 (7)	0.0213 (7)	0.0107 (6)	0.0007 (6)	−0.0018 (5)	0.0007 (5)
C11	0.0185 (7)	0.0181 (7)	0.0100 (6)	0.0035 (5)	−0.0012 (5)	0.0029 (5)
C12	0.0180 (7)	0.0249 (7)	0.0136 (6)	0.0062 (6)	0.0020 (5)	0.0058 (5)
C13	0.0265 (8)	0.0340 (8)	0.0152 (7)	0.0168 (7)	0.0041 (6)	0.0052 (6)
C14	0.0396 (10)	0.0197 (7)	0.0140 (7)	0.0109 (7)	0.0003 (6)	0.0019 (5)
C15	0.0260 (8)	0.0175 (7)	0.0126 (6)	0.0026 (6)	−0.0034 (5)	0.0022 (5)
C16	0.0278 (8)	0.0242 (8)	0.0151 (7)	0.0087 (6)	0.0049 (6)	0.0062 (6)
C17	0.0267 (8)	0.0247 (7)	0.0108 (6)	0.0031 (6)	0.0007 (5)	0.0018 (5)
C18	0.0255 (8)	0.0321 (8)	0.0123 (7)	0.0083 (7)	−0.0022 (6)	0.0052 (6)
C19	0.0346 (9)	0.0215 (7)	0.0155 (7)	0.0089 (6)	0.0020 (6)	0.0072 (5)
C20	0.0243 (8)	0.0269 (8)	0.0168 (7)	0.0011 (6)	0.0029 (6)	0.0085 (6)
C21	0.0144 (6)	0.0159 (6)	0.0114 (6)	0.0024 (5)	−0.0012 (5)	0.0019 (5)
C22	0.0168 (6)	0.0204 (7)	0.0121 (6)	0.0027 (5)	−0.0001 (5)	0.0027 (5)
C23	0.0181 (7)	0.0189 (7)	0.0180 (7)	0.0016 (5)	0.0017 (5)	0.0053 (5)
C24	0.0234 (8)	0.0285 (8)	0.0210 (7)	0.0043 (6)	0.0057 (6)	0.0060 (6)
C25	0.0138 (6)	0.0176 (6)	0.0089 (6)	0.0009 (5)	−0.0012 (4)	0.0032 (5)
C26	0.0171 (6)	0.0174 (6)	0.0131 (6)	0.0019 (5)	−0.0009 (5)	0.0011 (5)
C27	0.0250 (7)	0.0190 (7)	0.0155 (7)	0.0038 (6)	−0.0025 (5)	0.0031 (5)
C28	0.0224 (7)	0.0225 (7)	0.0244 (8)	0.0066 (6)	0.0003 (6)	0.0003 (6)

### Geometric parameters (Å, º)

**Table:**

Fe1—C2	2.0504 (14)	C9—H9A	0.9800
Fe1—C6	2.0507 (15)	C10—H10A	0.9800
Fe1—C3	2.0519 (15)	C11—C12	1.433 (2)
Fe1—C7	2.0531 (14)	C11—C15	1.433 (2)
Fe1—C8	2.0574 (15)	C11—C25	1.5055 (19)
Fe1—C9	2.0605 (15)	C12—C13	1.424 (2)
Fe1—C4	2.0609 (15)	C12—H12A	0.9800
Fe1—C10	2.0622 (14)	C13—C14	1.423 (2)
Fe1—C5	2.0634 (14)	C13—H13A	0.9800
Fe1—C1	2.0691 (13)	C14—C15	1.422 (2)
Fe2—C18	2.0450 (15)	C14—H14A	0.9800
Fe2—C15	2.0460 (14)	C15—H15A	0.9800
Fe2—C19	2.0486 (14)	C16—C17	1.423 (2)
Fe2—C14	2.0500 (15)	C16—C20	1.429 (2)
Fe2—C13	2.0513 (15)	C16—H16A	0.9800
Fe2—C17	2.0517 (15)	C17—C18	1.426 (2)
Fe2—C12	2.0546 (14)	C17—H17A	0.9800
Fe2—C20	2.0549 (15)	C18—C19	1.421 (2)
Fe2—C11	2.0572 (14)	C18—H18A	0.9800
Fe2—C16	2.0613 (16)	C19—C20	1.426 (2)
O1—C21	1.4399 (15)	C19—H19A	0.9800
O1—C25	1.4430 (15)	C20—H20A	0.9800
C1—C5	1.4306 (19)	C21—C22	1.5280 (18)
C1—C2	1.4349 (19)	C21—H21A	0.9800
C1—C21	1.5111 (18)	C22—C23	1.4962 (19)
C2—C3	1.429 (2)	C22—H22A	0.9700
C2—H2A	0.9800	C22—H22B	0.9700
C3—C4	1.429 (2)	C23—C24	1.327 (2)
C3—H3A	0.9800	C23—H23A	0.9300
C4—C5	1.427 (2)	C24—H24A	0.9300
C4—H4A	0.9800	C24—H24B	0.9300
C5—H5A	0.9800	C25—C26	1.5292 (18)
C6—C7	1.420 (2)	C25—H25A	0.9800
C6—C10	1.423 (2)	C26—C27	1.497 (2)
C6—H6A	0.9800	C26—H26A	0.9700
C7—C8	1.420 (2)	C26—H26B	0.9700
C7—H7A	0.9800	C27—C28	1.323 (2)
C8—C9	1.426 (2)	C27—H27A	0.9300
C8—H8A	0.9800	C28—H28A	0.9300
C9—C10	1.426 (2)	C28—H28B	0.9300
			
C2—Fe1—C6	113.59 (6)	C7—C6—H6A	126.1
C2—Fe1—C3	40.76 (6)	C10—C6—H6A	126.1
C6—Fe1—C3	141.86 (6)	Fe1—C6—H6A	126.1
C2—Fe1—C7	109.54 (6)	C8—C7—C6	108.42 (13)
C6—Fe1—C7	40.48 (6)	C8—C7—Fe1	69.96 (8)
C3—Fe1—C7	111.39 (6)	C6—C7—Fe1	69.67 (8)
C2—Fe1—C8	134.33 (6)	C8—C7—H7A	125.8
C6—Fe1—C8	68.19 (6)	C6—C7—H7A	125.8
C3—Fe1—C8	108.04 (6)	Fe1—C7—H7A	125.8
C7—Fe1—C8	40.41 (6)	C7—C8—C9	107.94 (14)
C2—Fe1—C9	174.21 (6)	C7—C8—Fe1	69.63 (8)
C6—Fe1—C9	68.28 (6)	C9—C8—Fe1	69.86 (8)
C3—Fe1—C9	134.47 (6)	C7—C8—H8A	126.0
C7—Fe1—C9	68.03 (6)	C9—C8—H8A	126.0
C8—Fe1—C9	40.52 (6)	Fe1—C8—H8A	126.0
C2—Fe1—C4	68.33 (6)	C10—C9—C8	107.67 (13)
C6—Fe1—C4	177.47 (6)	C10—C9—Fe1	69.83 (8)
C3—Fe1—C4	40.66 (6)	C8—C9—Fe1	69.62 (8)
C7—Fe1—C4	141.04 (7)	C10—C9—H9A	126.2
C8—Fe1—C4	111.81 (6)	C8—C9—H9A	126.2
C9—Fe1—C4	109.98 (6)	Fe1—C9—H9A	126.2
C2—Fe1—C10	144.29 (6)	C6—C10—C9	108.20 (13)
C6—Fe1—C10	40.47 (6)	C6—C10—Fe1	69.33 (8)
C3—Fe1—C10	174.93 (6)	C9—C10—Fe1	69.70 (8)
C7—Fe1—C10	67.82 (6)	C6—C10—H10A	125.9
C8—Fe1—C10	67.96 (6)	C9—C10—H10A	125.9
C9—Fe1—C10	40.47 (6)	Fe1—C10—H10A	125.9
C4—Fe1—C10	137.03 (6)	C12—C11—C15	106.83 (13)
C2—Fe1—C5	68.04 (6)	C12—C11—C25	126.74 (13)
C6—Fe1—C5	138.15 (6)	C15—C11—C25	126.26 (13)
C3—Fe1—C5	68.21 (6)	C12—C11—Fe2	69.50 (8)
C7—Fe1—C5	176.90 (6)	C15—C11—Fe2	69.13 (8)
C8—Fe1—C5	142.68 (6)	C25—C11—Fe2	129.98 (9)
C9—Fe1—C5	114.57 (6)	C13—C12—C11	108.61 (14)
C4—Fe1—C5	40.49 (6)	C13—C12—Fe2	69.58 (8)
C10—Fe1—C5	112.86 (6)	C11—C12—Fe2	69.70 (8)
C2—Fe1—C1	40.77 (5)	C13—C12—H12A	125.7
C6—Fe1—C1	111.73 (6)	C11—C12—H12A	125.7
C3—Fe1—C1	68.73 (6)	Fe2—C12—H12A	125.7
C7—Fe1—C1	136.39 (6)	C14—C13—C12	107.90 (13)
C8—Fe1—C1	174.97 (6)	C14—C13—Fe2	69.65 (9)
C9—Fe1—C1	144.45 (6)	C12—C13—Fe2	69.84 (8)
C4—Fe1—C1	68.51 (6)	C14—C13—H13A	126.1
C10—Fe1—C1	115.48 (6)	C12—C13—H13A	126.1
C5—Fe1—C1	40.51 (5)	Fe2—C13—H13A	126.1
C18—Fe2—C15	160.62 (7)	C15—C14—C13	108.08 (13)
C18—Fe2—C19	40.63 (7)	C15—C14—Fe2	69.53 (8)
C15—Fe2—C19	124.65 (6)	C13—C14—Fe2	69.74 (9)
C18—Fe2—C14	122.93 (6)	C15—C14—H14A	126.0
C15—Fe2—C14	40.63 (6)	C13—C14—H14A	126.0
C19—Fe2—C14	105.68 (6)	Fe2—C14—H14A	126.0
C18—Fe2—C13	105.42 (6)	C14—C15—C11	108.58 (14)
C15—Fe2—C13	68.39 (6)	C14—C15—Fe2	69.84 (8)
C19—Fe2—C13	118.15 (6)	C11—C15—Fe2	69.97 (8)
C14—Fe2—C13	40.61 (7)	C14—C15—H15A	125.7
C18—Fe2—C17	40.74 (6)	C11—C15—H15A	125.7
C15—Fe2—C17	157.60 (6)	Fe2—C15—H15A	125.7
C19—Fe2—C17	68.19 (6)	C17—C16—C20	107.58 (14)
C14—Fe2—C17	160.82 (7)	C17—C16—Fe2	69.39 (9)
C13—Fe2—C17	124.79 (7)	C20—C16—Fe2	69.44 (9)
C18—Fe2—C12	119.73 (6)	C17—C16—H16A	126.2
C15—Fe2—C12	68.30 (6)	C20—C16—H16A	126.2
C19—Fe2—C12	153.71 (6)	Fe2—C16—H16A	126.2
C14—Fe2—C12	68.21 (6)	C16—C17—C18	108.52 (14)
C13—Fe2—C12	40.58 (6)	C16—C17—Fe2	70.12 (9)
C17—Fe2—C12	108.74 (6)	C18—C17—Fe2	69.38 (8)
C18—Fe2—C20	68.52 (6)	C16—C17—H17A	125.7
C15—Fe2—C20	108.43 (6)	C18—C17—H17A	125.7
C19—Fe2—C20	40.68 (6)	Fe2—C17—H17A	125.7
C14—Fe2—C20	119.83 (7)	C19—C18—C17	107.66 (14)
C13—Fe2—C20	153.74 (7)	C19—C18—Fe2	69.82 (9)
C17—Fe2—C20	68.18 (6)	C17—C18—Fe2	69.88 (8)
C12—Fe2—C20	164.60 (6)	C19—C18—H18A	126.2
C18—Fe2—C11	155.89 (7)	C17—C18—H18A	126.2
C15—Fe2—C11	40.89 (6)	Fe2—C18—H18A	126.2
C19—Fe2—C11	162.99 (7)	C18—C19—C20	108.30 (14)
C14—Fe2—C11	68.73 (6)	C18—C19—Fe2	69.55 (9)
C13—Fe2—C11	68.77 (6)	C20—C19—Fe2	69.90 (8)
C17—Fe2—C11	122.23 (6)	C18—C19—H19A	125.9
C12—Fe2—C11	40.80 (5)	C20—C19—H19A	125.9
C20—Fe2—C11	126.96 (6)	Fe2—C19—H19A	125.9
C18—Fe2—C16	68.56 (7)	C19—C20—C16	107.94 (15)
C15—Fe2—C16	122.45 (7)	C19—C20—Fe2	69.42 (9)
C19—Fe2—C16	68.37 (6)	C16—C20—Fe2	69.93 (9)
C14—Fe2—C16	156.05 (8)	C19—C20—H20A	126.0
C13—Fe2—C16	162.95 (8)	C16—C20—H20A	126.0
C17—Fe2—C16	40.49 (6)	Fe2—C20—H20A	126.0
C12—Fe2—C16	127.34 (6)	O1—C21—C1	110.91 (10)
C20—Fe2—C16	40.63 (6)	O1—C21—C22	103.96 (10)
C11—Fe2—C16	109.82 (6)	C1—C21—C22	113.26 (11)
C21—O1—C25	116.26 (10)	O1—C21—H21A	109.5
C5—C1—C2	106.88 (12)	C1—C21—H21A	109.5
C5—C1—C21	126.00 (13)	C22—C21—H21A	109.5
C2—C1—C21	127.05 (12)	C23—C22—C21	113.86 (11)
C5—C1—Fe1	69.53 (8)	C23—C22—H22A	108.8
C2—C1—Fe1	68.92 (8)	C21—C22—H22A	108.8
C21—C1—Fe1	129.00 (9)	C23—C22—H22B	108.8
C3—C2—C1	108.67 (13)	C21—C22—H22B	108.8
C3—C2—Fe1	69.68 (8)	H22A—C22—H22B	107.7
C1—C2—Fe1	70.32 (8)	C24—C23—C22	123.85 (14)
C3—C2—H2A	125.7	C24—C23—H23A	118.1
C1—C2—H2A	125.7	C22—C23—H23A	118.1
Fe1—C2—H2A	125.7	C23—C24—H24A	120.0
C2—C3—C4	107.79 (13)	C23—C24—H24B	120.0
C2—C3—Fe1	69.56 (8)	H24A—C24—H24B	120.0
C4—C3—Fe1	70.00 (8)	O1—C25—C11	110.74 (10)
C2—C3—H3A	126.1	O1—C25—C26	104.26 (10)
C4—C3—H3A	126.1	C11—C25—C26	113.26 (11)
Fe1—C3—H3A	126.1	O1—C25—H25A	109.5
C5—C4—C3	107.79 (13)	C11—C25—H25A	109.5
C5—C4—Fe1	69.85 (8)	C26—C25—H25A	109.5
C3—C4—Fe1	69.33 (8)	C27—C26—C25	113.16 (11)
C5—C4—H4A	126.1	C27—C26—H26A	108.9
C3—C4—H4A	126.1	C25—C26—H26A	108.9
Fe1—C4—H4A	126.1	C27—C26—H26B	108.9
C4—C5—C1	108.87 (13)	C25—C26—H26B	108.9
C4—C5—Fe1	69.66 (8)	H26A—C26—H26B	107.8
C1—C5—Fe1	69.96 (8)	C28—C27—C26	123.68 (14)
C4—C5—H5A	125.6	C28—C27—H27A	118.2
C1—C5—H5A	125.6	C26—C27—H27A	118.2
Fe1—C5—H5A	125.6	C27—C28—H28A	120.0
C7—C6—C10	107.77 (13)	C27—C28—H28B	120.0
C7—C6—Fe1	69.85 (8)	H28A—C28—H28B	120.0
C10—C6—Fe1	70.20 (8)		
